# ALGI: Sparse Convolutional Denoising Autoencoder Utilizing Local Genomic Information for Genotype Imputation

**DOI:** 10.3390/ani16111588

**Published:** 2026-05-23

**Authors:** Taotao Tan, Bingxi Gao, Rong Zhang, Huaxuan Wu, Zongjun Yin, Cai-Xia Yang, Zhi-Qiang Du

**Affiliations:** 1 College of Animal Science and Technology, Yangtze University, Jingzhou 434020, China; tantt924@163.com (T.T.); gbx15020771250@163.com (B.G.); 202071757@yangtzeu.edu.cn (R.Z.); 2021710855@yangtzeu.edu.cn (H.W.); caixiayang@yangtzeu.edu.cn (C.-X.Y.); 2 College of Animal Science and Technology, Anhui Agricultural University, Hefei 230036, China; yinzongjun@ahau.edu.cn

**Keywords:** genotype imputation, K-means, autoencoder, deep learning, local genomic, convolution, genotype inference, Beagle

## Abstract

Genotype imputation (GI) plays a critical role in predicting missing genetic information for genomic studies and breeding applications. Although recent reference-free deep learning approaches have demonstrated promising performance, they often fail to exploit local genomic information, which limits further improvements in prediction accuracy and stability. In this study, we developed ALGI, a novel method based on a sparse convolutional denoising autoencoder, which uniquely integrates local genomic window information with group-specific feature learning. Unlike conventional convolutional or autoencoder-based approaches, ALGI first applies K-means clustering to group samples according to local genomic windows, then learns hidden genotype configurations specific to each group, capturing fine-scale local patterns and complex haplotype structures. Systematic evaluation was conducted across yeast, human, and pig MHC regions under multiple scenarios, including different window sizes, missing rates, sample sizes, and numbers of variants. Results show that ALGI demonstrates consistent improvements over conventional methods (Beagle) and state-of-the-art deep learning approaches (AE, SCDA) under the evaluated settings, with enhanced accuracy, stability, and robustness. In addition, ALGI is user-friendly and publicly available. While evaluated on highly polymorphic MHC regions, its strong performance suggests applicability to less complex regions, though broader genome-wide validation is needed. This approach provides a powerful tool for genomic selection and advancing complex trait genetics in livestock and other species.

## Simple Summary

Genotype imputation (GI) is widely used to predict missing genetic information, which is essential for genomic studies and breeding programs. Recent deep learning approaches have shown promising results without relying on reference panels, but they often overlook important local genomic patterns that could improve prediction accuracy. In this study, we developed a novel method called ALGI (sparse convolutional denoising autoencoder with local genomic information), which integrates local genomic information into a deep learning framework. By grouping samples based on local genomic windows and applying a sparse convolutional denoising autoencoder, the model effectively captures complex genetic structures and improves the accuracy and stability of genotype imputation. This approach provides a more reliable and efficient solution for genomic data analysis, supporting advances in precision breeding and genetic research.

## 1. Introduction

Genotype imputation (GI) refers to the inference of missing genotypes based on phased haplotypes for a reference panel (a large collection of samples), or directly from the observed data without relying on an external reference panel [1,2,3,4]. Missing genotypes can arise due to experimental or technical limitations in different genome resequencing or microarray platforms, for instance, low-coverage genome resequencing projects. By inferring and integrating genotype data from different projects or resources, GI can boost the statistical power of genome-wide association study (GWAS), and identification of causal variants (fine-mapping), for complex trait genetics in both agricultural production and human disease studies [5,6,7,8]. Furthermore, GI can be applied for a variety of other purposes, such as gene–environment interaction analysis [9], detection of selection signature or footprint [10], and reconstruction of ancient and private genomes [11,12].

Factors affecting GI performance include the demographic structure and evolutionary history of the reference panel [3], the number and coverage of genomic variants and recombination events within the genomic region [13], and the frequency of rare alleles [14]. The linkage disequilibrium (LD) between genetic variants is fundamental to GI performance. The simpler the LD structure, the higher the imputation accuracy. Reference panels with similar genetic and evolutionary backgrounds tend to exhibit less complex LD patterns [15,16]. Based on the pre-phased haplotype panels, missing genotypes are imputed using various statistical or population genetics models, such as haplotype clustering [3], the hidden Markov model (HMM) [4,17], or the coalescent model [18]. Multiple large and representative reference panels have been developed for both animal and human populations [19,20,21], along with imputation pipelines and servers [22,23,24], to improve GI efficiency and accuracy. Moreover, algorithm optimization and parallelization were adopted to enhance the efficiency of reference-based phasing and imputation, preparing for future reference panels on the scale of one million human genomes [25,26]. For non-model organisms, especially in animal genetics and breeding, numerous tools and platforms have also been developed [5,8,27,28].

In addition to conventional statistical methods for GI [2,3,17], machine learning approaches have also been applied, including mean imputation, singular value decomposition (SVD), K-nearest neighbor (KNN) and random forest. These methods are widely used in reference panel construction [29], quality calibration (MagicalRsq) [30], and scalability improvement [31]. Recently, due to its strength in dealing with large-scale datasets and highly complex non-linear relationships, the deep learning method has also been applied in GI. Moreover, deep learning methods have shown advantages in capturing complex non-linear relationships and can operate without relying on reference panels. Despite their potential, these methods do not reliably outperform traditional statistical approaches; in many cases, deep learning provides only modest improvements in accuracy, particularly under specific data conditions or population structures [32,33,34,35,36]. Autoencoders (AE) have been proposed to process input data by imposing constraints on network architecture, enabling dimensionality reduction, denoising, and demasking in GI [37]. By combining the advantages of AE and convolutional neural networks (CNN), the sparse convolutional denoising autoencoder (SCDA) can effectively extract the local relationships embedded in genotype data and accurately infer the missing genotypes [38]. However, the SCDA model has several limitations: it is not designed for multiple inputs and outputs; its training process relies on default mini-batch average loss, and predefined training cycles cannot be fully implemented, which restricts further performance improvement. Furthermore, to enhance the performance of deep learning-based GI methods, recombination information within local genomic regions should be considered [39,40,41]. Local ancestral diagnosis provides better resolution and precision for genetic kinship and complex trait genetics analyses [11,42].

However, existing genotype imputation methods, especially reference-free deep learning approaches, often overlook local genomic structures such as LD patterns and haplotype configurations, limiting further improvements in accuracy and robustness. To address this limitation, grouping samples based on local genomic patterns can reduce intra-group heterogeneity, as samples within local regions often share similar linkage disequilibrium (LD) and haplotype structures. This provides a more consistent input for deep learning models and facilitates more accurate genotype inference. Here, we developed the sparse convolutional denoising autoencoder on local genomic information for GI (ALGI), through first clustering samples into different groups based on local genomic information, and then decomposing the genetic relatedness and haplotype configurations embedded in local genomic regions by autoencoder, to improve GI performance. Using yeast, human, and pig SLA genotype data from complex genomic regions, ALGI was further evaluated and shown to outperform multiple state-of-the-art methods across different analytical scenarios.

## 2. Materials and Methods

### 2.1. ALGI Framework

**Figure 1:**
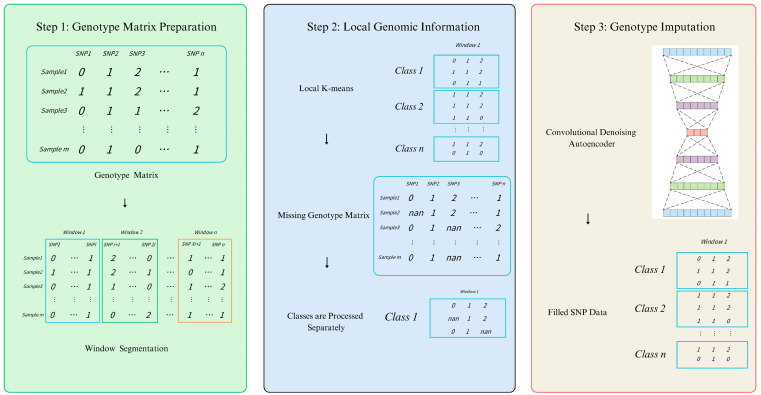
Schematic diagram of the ALGI genotype imputation model.

The ALGI framework consisted of three major components: SNP genotype data processing and matrix construction (including random generation of missing values), preparation of different batches of local genome windows and sample clustering, and utilizing the deep learning SCDA model for GI (Figure 1).

The different colored sections in Step 1 and Step 3 represent different windows and different network layers, respectively. The encoder consisted of two Conv1D layers (128 and 64 filters, kernel size = 3, ReLU activation), each followed by max-pooling (pool size = 2), with dropout and L1 regularization applied to reduce overfitting. The decoder mirrored this structure using up-sampling layers and Conv1D layers (64 and 128 filters). The final output layer uses a Softmax activation to reconstruct genotype probabilities across three states. The model was trained using the Adam optimizer (learning rate = 0.001) with categorical cross-entropy loss (Table A1). Full implementation details are available in our GitHub v2.1 repository (https://github.com/Bingxi-Gao/ALGI2, accessed on 12 May 2026).

### 2.2. Datasets

Three single nucleotide polymorphism (SNP) genotype datasets: yeast, human, and pig (Table 1) [34,43,44], were used, mainly to demonstrate the inference performance of ALGI, specifically on the major histocompatibility complex (MHC) regions for human and pig (or leukocyte antigen systems, HLA, and SLA). HLA and SLA regions show high diversity and heterogeneity, and are associated with many complex diseases. The yeast and HLA genotype datasets have a relatively large number of SNPs, 28,220 and 27,209, respectively. The yeast data come from 3513 strains, isolated from a vineyard, and cultured in the laboratory [44]. HLA genotype data (on human chromosome 6) cover 2504 individuals from five different ethnic groups (American, European, African, East, and South Asian) [34,43]. SLA (on pig chromosome 7) data include 2549 animals, and a relatively small number of SNPs (2173) [43]. The datasets were publicly available, as described in the corresponding references.

Furthermore, we evaluated a Type 2 diabetes (T2D) dataset with a relatively small sample size, consisting of 92 cases with a family history of T2D and 93 controls without T2D history, to explore the potential reasons for the low accuracy of genotype imputation (GI) and its implications for fine mapping [18].

### 2.3. Genotype Matrix Construction

**Figure A1:**
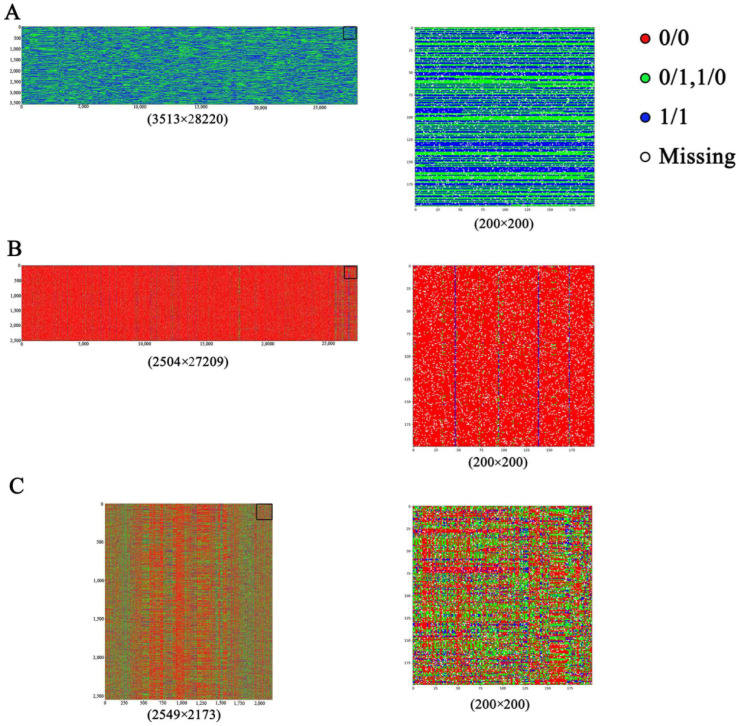
Visualization of genotype data for yeast (**A**), HLA (**B**), and SLA (**C**) (missing 10% data).

First, for each dataset, genotype matrices for all samples and SNP markers were built, in which all SNP genotypes were replaced (“0/0” to “0”, “1/0” or “0/1” to “1”, and “1/1” to “2”). In addition, missing genotypes were randomly generated (changed to “3”) These values serve only as categorical labels rather than quantitative measures, and facilitate subsequent one-hot encoding for model input. Then, as one-hot encoding was applied, “0” was changed to [1, 0, 0, 0], “1” to [0, 1, 0, 0], “2” to [0, 0, 1, 0] and “3” to [0, 0, 0, 1]. After deleting the last dimension, that is, “0” to [1, 0, 0], “1” to [0, 1, 0], “2” to [0, 0, 1] and “3” to [0, 0, 0], missing and real genotypes can be distinguished. Genotype matrices for all three datasets were plotted and visualized, to evaluate data heterogeneity by picture entropy calculation (Figure A1).

### 2.4. Local Genome Window Batches and Sample Clustering

Second, to better extract the hidden features embedded in local genome regions, we divided the genome window into batches with different sizes (number of SNPs), ranging from 100 to 1000 SNPs per batch. Next, for each batch, K-means clustering was applied on the genotype matrix in the local region, to better cluster samples by utilizing the local genetic relationship. The clustering and window construction were performed separately within the training data to prevent any information leakage into the validation and test sets. Since genome sub-regions within the same population can have different evolutionary and genetic histories, local clustering can more accurately capture relationships among samples, thereby improving downstream genotype imputation performance [38,39].

To ensure that each class had sufficient samples for subsequent data partitioning and model training, we modified the traditional K-means clustering algorithm. Briefly, preliminary clustering was performed by K-means clustering, by defining the number of clusters first, to obtain the clustering center and labels. Then, the number of samples in each cluster was checked, to be no less than the pre-defined number of samples (e.g., 20). If a cluster had a limited number of samples, adjustments were performed as follows: find the clusters with sufficient and insufficient number of samples, and reassign the samples from the sufficient cluster closest to the center of the insufficient cluster, until all clusters have the predefined number of samples. The modification ensures a minimum number of samples per cluster for stable model training. We explicitly note that sample reassignment may introduce artificial structure, but the impact is minimal and constrained to preserve local similarity.

### 2.5. Sparse Convolutional Denoising Autoencoder (SCDA)

Lastly, SCDA was employed to decompose the local genotype matrix information, in order to infer and fill in the missing genotype. Here, SCDA was designed to be composed of seven layers, one input layer and six convolutional layers. The input layer accepted the one-hot encoded genotype matrix, and the data dimension was (None, 3), in which None represented the input sequences of variable length. At the encoding stage, CNN was used to extract the data features. Briefly, the first convolutional layer was equipped with 128 filters (size 3), and the ReLU activation function and padding were applied to keep the input data dimension unchanged. Then, max pooling (window size 2) was used to reduce the data dimension. The second convolutional layer used 64 filters, and also ReLU and max pooling. Using a multi-step encoding process that included six convolutional layers, the genotype matrix was efficiently compressed into a low-dimensional embedding space.

During decoding, the decoder architecture was constructed to correspond to the encoder, enabling the projection of the compressed feature representation into the original input domain. Data reconstruction was achieved using several convolutional layers, up sampling operations, and suitable activation functions such as Softmax.

In addition, in the convolutional layers, L1 regularization was introduced to help reduce the model complexity, by penalizing on the absolute value of weight coefficients, and to make the model more sparse in the training process [38]. The sparsity constraint encourages the model to learn more compact and informative latent representations by activating only a subset of neurons. This strategy was important to capture the highly correlated LD patterns in the genotype matrices.

To mitigate overfitting, a dropout rate of 25% was employed, randomly removing neurons and their associated connections to enhance generalization. The loss function was set to categorical_crossentropy for the multi-class classification task. Model parameters were optimized using Adam with suitably chosen learning rates to ensure effective weight updates throughout training.

### 2.6. Model Training and Evaluation

Three datasets used in the present study were processed independently to effectively extract the hidden data information. Each dataset was divided into training, validation, and testing data (64%:16%:20%). To evaluate the model performance on different data scale and missing rates, three missing rates of genotypes (5%, 10%, and 20%) were set on the three datasets, and missing genotypes were randomly generated. The model was evaluated, and results were obtained on 10 replicates. Genotype accuracy was calculated as the proportion of correctly imputed genotypes among all evaluated loci, i.e., the number of correctly predicted genotypes divided by the total number of masked genotypes (Equation (1)).

(1) $$Accuracy=\frac{1}{N}\sum_{i=1}^{N}1\left(y_{i}={\hat{y}}_{i}\right)$$

where N is the total number of evaluated (masked) genotypes, $y_{i}$ is the true genotype of the i-th locus, ${\hat{y}}_{i}$ is the imputed genotype, and $1\left(\cdot \right)$ is an indicator function that equals 1 if $y_{i}={\hat{y}}_{i}$ and 0 otherwise.

### 2.7. Hyperparameter Optimization

The ALGI model was constructed using the TensorFlow v1.13.1 framework. The hyperparameters were adjusted by 10 replicate experiments (Table A1). The start value of the learning rate was set to 0.001. When the learning rate was initiated with a small value, the training procedure converged slowly and required more epochs (a period of computing time) to reach a better local minimum. In contrast, when the learning rate was large, the training procedure oscillated around, but could not reach, the local optimum. The Adam optimizer was chosen in this study. Mini-batch gradient descent offered computational efficiency, and improved convergence compared to processing each sample individually (stochastic gradient descent) or the entire dataset at once (batch gradient descent). The batch-size was set to 32, i.e., 32 samples were extracted from the training samples for training each time (one batch), and the gradient was updated. The numbers of epochs were set at 50, 100, and 140 for yeast, HLA, and SLA, respectively. The hardware configuration was set up as: Intel^®^ Xeon^®^ CPU E7-4820 v4 @ 2.00 GHz; 48 G RAM; RTX3090 GPU; Python 3.8.0 and Cuda 11.4.

### 2.8. Overfitting Mitigation

Early stopping was employed as a regularization technique in deep learning to avoid overfitting. It involved monitoring the model’s performance on a validation dataset during training, and stopping the training process when the performance started to degrade. By preventing the model from learning noise in the training data, early stopping helped generalize better to the unseen data. It prevents overfitting to the new dataset, while still allowing the model to capture relevant patterns, thus improving performance on the new task.

### 2.9. Model Performance Evaluation

Conventional statistical methods (Beagle 5.4) [4,45,46] run in a reference-free mode, and state-of-the-art deep learning methods (SCDA, AE) [37,38] on genotype imputation were compared with ALGI. Different scenarios were considered, including K-values of K-means clustering, window batch size (number of SNPs: 100 to 1000, with a step of 100), missing rates of genotype data (5%, 10%, and 20%), and number of samples (200, 500, 1000, 2000, and all). Then GI accuracy comparisons between different scenarios were made to assess the model performance.

To evaluate the stability of the model, the process was repeated 10 times, and the average and standard deviation of genotype accuracy in each result were recorded. This method is used to assess the robustness of the model by testing its accuracy and consistency across different subsets of the data.

## 3. Results

### 3.1. ALGI Model Evaluation

**Figure 2:**
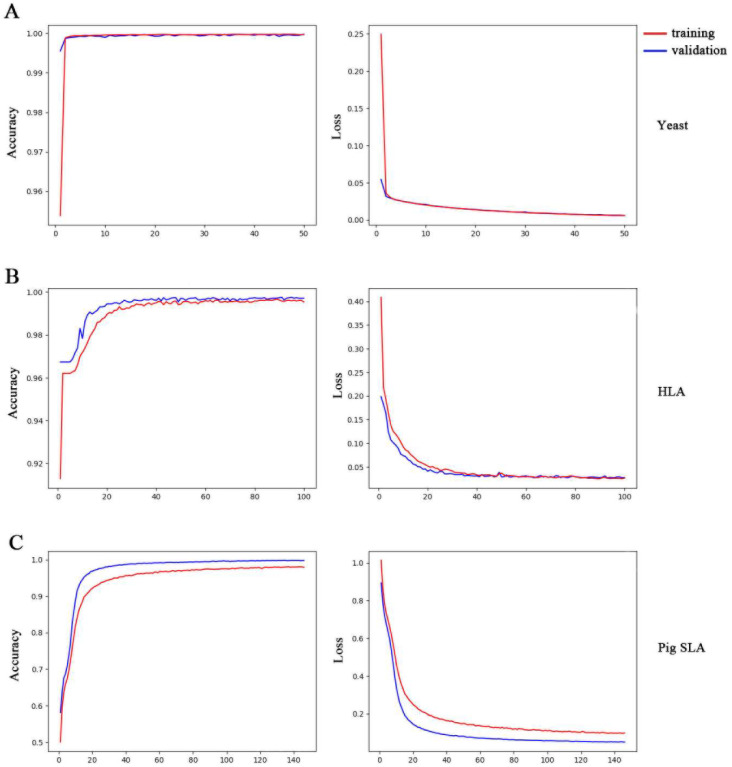
Accuracy and loss curves of training and validation processes of ALGI model. From top to bottom, (**A**) yeast dataset; (**B**) human leukocyte antigen (HLA) dataset; (**C**) swine leukocyte antigen (SLA) dataset. Genotype missing rate: 10%.

The hyperparameters of the ALGI model were first searched and optimized (details in Table A1). Then, the accuracy and losses along with different epochs during the ALGI model training and validation processes were examined (Figure 2). Overall, it was shown that the ALGI model was able to reach the stabilized state. For the yeast data, the model reached stable accuracy and loss levels within the first few epochs (Figure 2A). For the human HLA data, the model’s accuracy and loss stabilized at around 10 epochs—the elbow point (Figure 2B). However, for the pig SLA data, at least 20 epochs (the elbow point) were needed, possibly due to higher data heterogeneity or complexity (picture entropy calculation: 1.5856, 0.9907, and 1.8872 for yeast, HLA, and SLA, respectively) (Figure 2C).

### 3.2. ALGI Performs the Best in GI Accuracy

**Figure 3:**
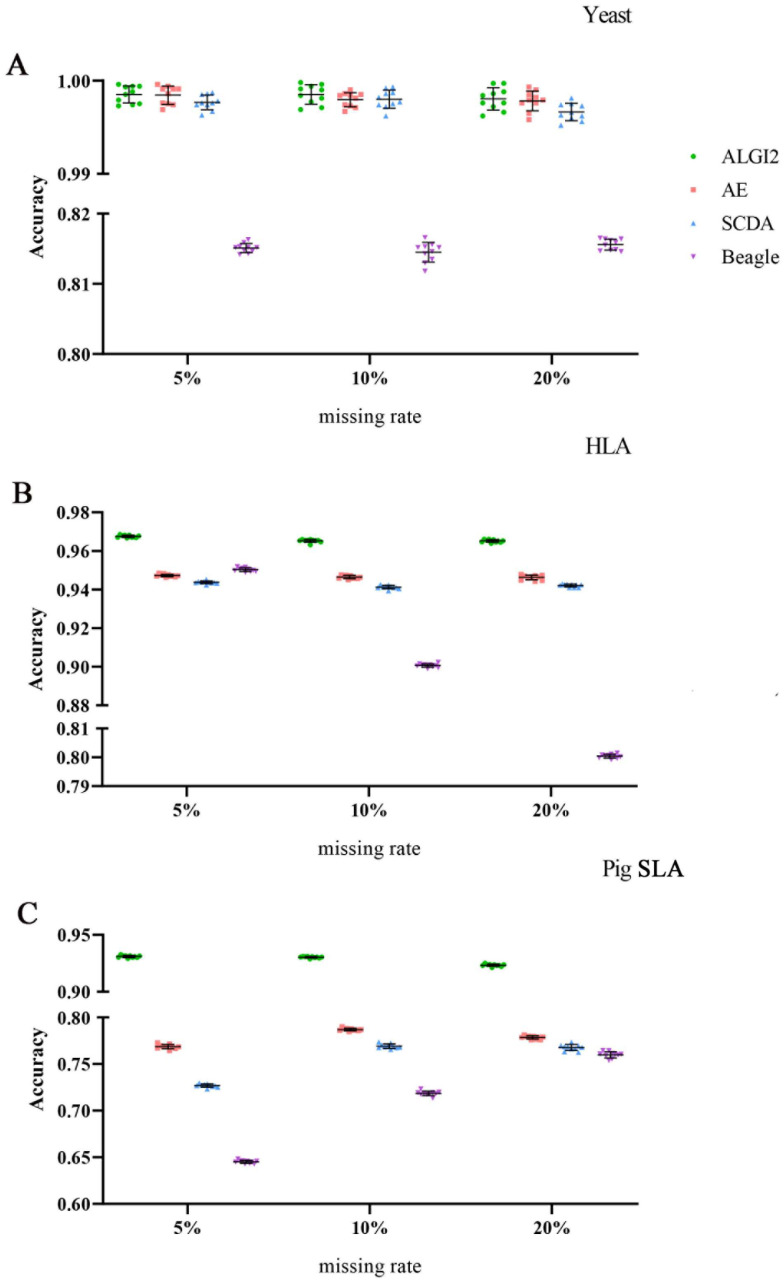
Violin plots of model performances for ALGI (green), SCDA (blue), AE (red), and Beagle (purple). Genotype missing rates: (**A**) 5%; (**B**) 10%; (**C**) 20%. The black lines indicate the variance interval around the mean.

**Figure A2:**
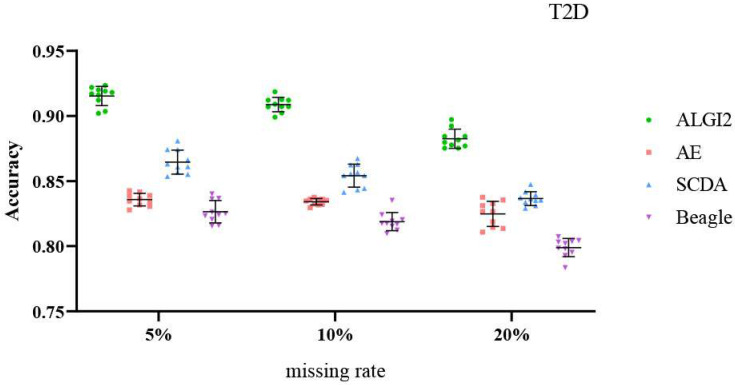
Performance of different models on T2D dataset. The black lines indicate the variance interval around the mean.

Genotype inference accuracy was compared between ALGI and several other models (AE, SCDA, and Beagle). Compared with SCDA and Beagle, ALGI achieved significantly higher genotype inference accuracy (paired sample *t*-test, all *p* < 0.05) across all three datasets and missing rates. ALGI also outperformed SCDA and showed significant advantages in HLA and pig SLA datasets (paired sample *t*-test, all *p* < 0.05), though ALGI remained the best overall. First, three different missing rates (5%, 10%, and 20%) were considered, by random genotype deletion on all three datasets (yeast, HLA, and SLA), and the average accuracy and standard deviations were calculated for 10 replicates (Figure 3). Compared to AE, SCDA, and Beagle, ALGI performed the best in genotype prediction accuracy, whereas Beagle had the worst performance. All models performed better on the yeast and HLA data, which exhibited tighter intra-group clustering compared with the pig SLA dataset (Figure A2). However, even on the more heterogeneous SLA data, ALGI still inferred genotypes with higher accuracy and consistency (Figure 3).

To further understand why ALGI performed better than AE and SCDA on the pig SLA data, two additional comparisons were done. First, on the HLA dataset, 2000 SNPs were randomly selected to set the number of markers similar to the pig SLA data. The model comparison was repeated to see if the number of SNP markers affected the prediction accuracy. However, the performance of AE and SCDA were not significantly affected (paired sample *t*-test, *p* > 0.05) (Table A2). In contrast, the performance of ALGI showed a noticeable decrease under the same condition, which may be due to the reduced genomic window limiting its ability to capture haplotype features. Next, to investigate whether chromosome linkage disequilibrium could be one of the influencing factors, 2000 SNPs on pig chromosome 1 were randomly chosen to simulate SLA data on pig chromosome 7. No significant differences among ALGI, AE, and SCDA were observed across chromosomes (paired-sample *t*-test, *p* > 0.05; Table A3). This suggests that the heterogeneity of the pig SLA data may underlie the lower genotype-prediction accuracy observed for all models.

### 3.3. Factors Affecting ALGI Performance

**Figure 4:**
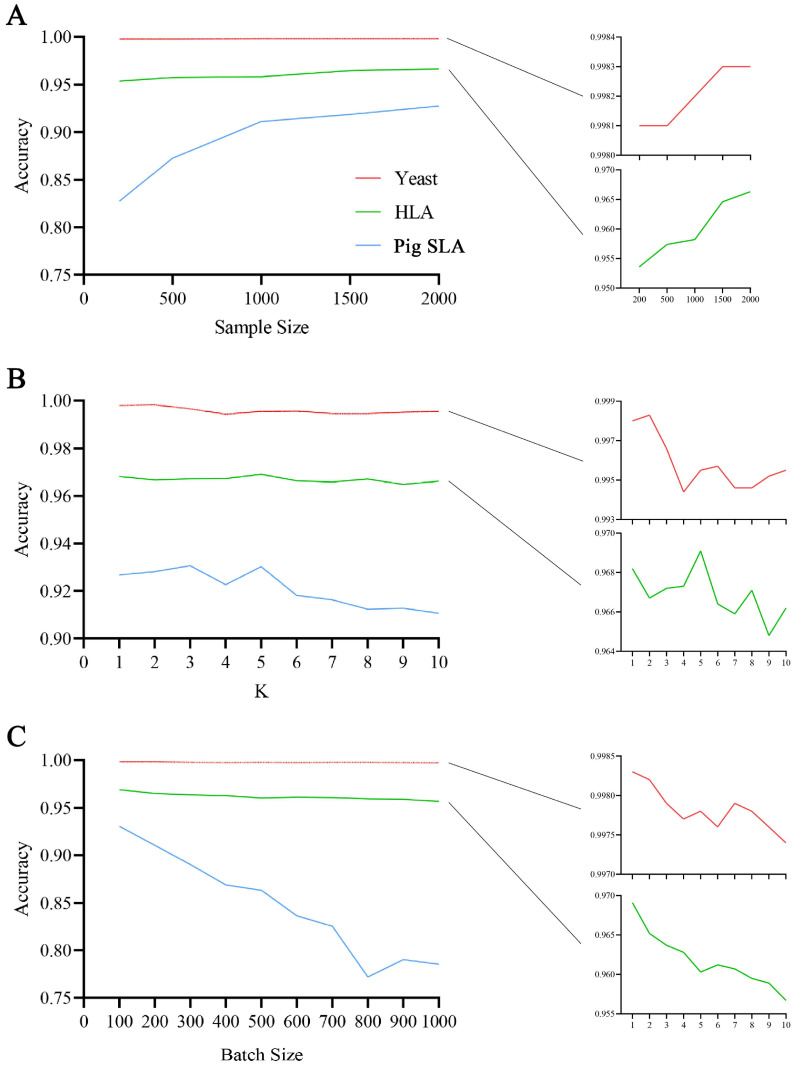
Prediction accuracy of three different scenarios (10% missing data rate). (**A**) Number of samples; (**B**) K-values; (**C**) Number of SNPs in each batch.

We further assessed the model performance and robustness of ALGI, based on 10% genotype missing rates, since similar results were obtained for 5% and 20% missing rates, as shown previously (Figure 3). Then, three different scenarios, regarding the number of samples, the K-values of K-means clustering, and the number of SNPs in window batches, were examined (Figure 4). We found that across all scenarios, the models achieved the best performance on the yeast dataset, followed by the HLA dataset, with the lowest performance on the pig SLA dataset..

First, the effect of sample size on prediction accuracy was evaluated. For each of the three datasets, samples were randomly selected to create five subsets of different sizes (200, 500, 1000, 1500, and 2000). As the sample size increased, the accuracy of ALGI improved, rising from 0.83 to 0.92 for the pig SLA data, whereas only slight gains were observed for the yeast (0.9981–0.9983) and HLA datasets (0.953–0.967) (Figure 4A and insets). These results indicate that larger sample sizes provide more informative genomic or haplotype configurations, thereby enabling more accurate genotype prediction (Figure 4A).

Next, another key parameter for ALGI, the K-value of K-means clustering, was assessed (the number of SNPs set at 100 for each window batch, and missing rate at 10%). ALGI had the highest prediction accuracy when K-values were 2 (yeast) and 5 (HLA), respectively. For the pig SLA data, a K-value of 3 yielded the best performance (Figure 4B). This suggests that selecting the appropriate number of clusters can improve genotype imputation in ALGI.

**Figure 5:**
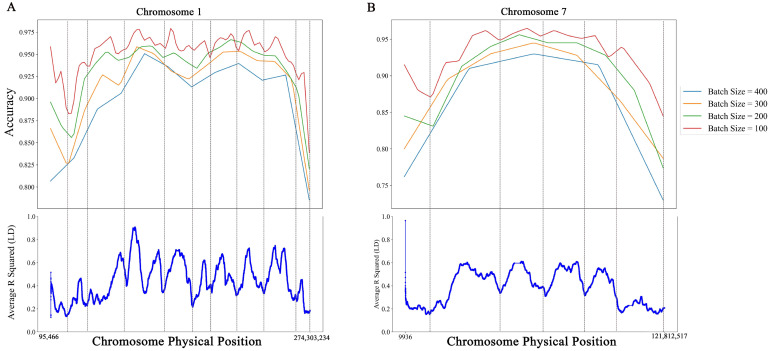
Effect of batch size on prediction accuracy and its relationship with LD status on pig SLA. (**A**) Chromosome 1; and (**B**) chromosome 7. Four different batch sizes (100, 200, 300, and 400) were considered. The blue line indicates the linkage disequilibrium state. Prediction accuracy correlates well with LD pattern on each chromosome.

Finally, we evaluated the effect of SNP window size (100–1000 SNPs, in steps of 100). As the window size increased, prediction accuracy declined gradually for the yeast data (≈0.99) and for the HLA data (0.969 to 0.957), but dropped sharply for the more heterogeneous pig SLA data (0.93 to 0.77) (Figure 4C). Though more SNPs increase the amount of information, a large genomic region introduces more uncertainty of haplotype configuration, and decreases the genotype imputation accuracy. To further demonstrate our point, we examined the relationship between LD status and window batch sizes, and the effect on genotype prediction accuracy (Figure 5). Two pig chromosomes (1 and 7) were considered, and batch sizes were set at four intervals (100, 200, 300, and 400). Again, batch size 100 had the highest prediction accuracy, and with the increase in batch sizes, the accuracy decreased (Figure 5A,B). In addition, genotype prediction accuracy was consistent with the dynamic change in LD pattern. The higher the LD in each region, the better the prediction accuracy. Thus, the accuracy of the ALGI model can also provide information on the local genomic LD status, and vice versa.

## 4. Discussion

A new reference-free deep learning model ALGI, combining sparse convolutional denoising autoencoder with local genomic information, was constructed and demonstrated to improve the accuracy of genotype imputation, better than traditional (Beagle) and other state-of-the-art deep learning methods (AE and SCDA). Sample size, window batch size and K-values (K-means clustering) were demonstrated to affect ALGI performance.

Traditional statistical methods and deep learning methods performed relatively accurately when dealing with less complex genetic data, as demonstrated on yeast and human data. However, for the pig data, they had relatively poor performance. SCDA and AE deep learning models largely work though the (convolutional) multi-layers, effectively extracting the hidden patterns embedded in genetic data, and realizing the sparse architecture through L1 regularization on the weighting matrix [7,10]. However, we further showed that through the utilization of local genomic information [19,22,38,39], more accurate haplotype information could be extracted by ALGI, and further improved the genotype imputation accuracy.

The accuracy of genotype imputation is affected by a variety of factors, such as the reference panel [9], number of variants and recombination events [3], and frequency of rare alleles [32]. In fact, all these factors affect the LD status (or haplotype configuration) between genetic variants that is fundamental to the performance of genotype imputation. Accurate phased haplotype information (with or without reference panels) is vital to genotype imputation [13]. ALGI improved the genotype imputation accuracy by utilizing local genomic/haplotype information and clustering samples into different classes. In addition, number of genetic variants in the window batches could affect the imputation accuracy, as demonstrated here, where pig SLA data had lower accuracy due to the relatively complex or heterogeneous haplotypes defined in the window batches. This increased heterogeneity in the pig SLA dataset may be driven by specific genomic features of the SLA region, including high levels of gene duplication, structural variation, and more complex haplotype structures, which can complicate the modeling of linkage disequilibrium and reduce imputation accuracy. The bigger the window batch size, the more recombination events, which can break down the linkage disequilibrium, and negatively affect the performance of genotype imputation.

Genotype imputation is important to fine mapping and causal variant discovery [24,26]. However, recently, concerns arose over genotype imputation accuracy, and its effect on fine mapping disease causal variants [18]. The authors suggested that the reduced performance may be caused by genetic variants absent from the reference panel. In addition, regardless of whether the density of genotyped SNPs is high or low, false-positive or false-negative associations may still be introduced. We further speculate that the limited sample size of the T2D dataset (only 185 samples and 197 SNPs) may also contribute to the reduced accuracy and stability of genotype prediction. However, ALGI still achieved the highest accuracy—0.9153, 0.9086, and 0.8824 at 5%, 10%, and 20% missing rates—outperforming both SCDA and AE, with Beagle performing the worst (Figure A2). Increasing the number of samples and genetic variants could further enhance ALGI’s performance.

Furthermore, the continuing evolution of deep learning technologies is expected to contribute to further improvements in prediction accuracy and usability in the future. ALGI was mainly designed for genotype imputation. However, the strategy can also be used to infer missing data in any data matrix, especially the high-dimensional matrix, and also other types of missing data, such as gene expression or DNA methylation data. In addition, ALGI can be extended to multi-task data imputation, such as multi-omics data integration and sequence imputation at the genome level, providing a potential framework and new perspectives for related studies, including genome-wide association studies, genomic selection, and polygenic risk prediction.

While K-means clustering provides a computationally efficient way to group samples based on local genomic similarity, it may not fully capture complex or non-linear genetic structures. Alternative methods, such as hierarchical clustering, Gaussian mixture models, or density-based approaches, could provide more flexible representations, although often at the cost of increased computational complexity and additional parameter tuning. Importantly, the ALGI framework is not restricted to K-means and can be extended to incorporate other clustering strategies.

Despite the promising performance of ALGI, several limitations should be noted. First, missing genotypes were simulated randomly, which may not fully reflect real-world missingness patterns. Second, the evaluation was primarily conducted on specific genomic regions (e.g., MHC), which may limit the generalizability of the findings to genome-wide scenarios. Third, the clustering strategy introduces certain assumptions, and although designed to reduce local heterogeneity, it may still introduce potential biases. Fourth, the speed and memory usage of ALGI was not evaluated. Additionally, Beagle was run in reference-free mode, without using an external reference panel, which may have contributed to its lower performance and represents a limitation in the comparative analysis. Furthermore, in real-world scenarios, missingness is often structured rather than random (e.g., due to platform-specific marker failures), and while this was not explicitly evaluated here, ALGI’s ability to model local genomic dependencies suggests it may be robust to certain structured missing patterns. These limitations should be addressed in future work through more realistic data settings, broader genomic evaluations, and exploration of alternative clustering strategies.

## 5. Conclusions

The ALGI framework improves genotype imputation accuracy by integrating a sparse convolutional denoising autoencoder (SCDA) with local genomic information. It outperforms existing methods in the evaluated genomic regions, particularly in complex loci such as the MHC. While these results demonstrate enhanced accuracy and robustness under the tested conditions, broader genome-wide validation is needed to confirm general applicability. In addition, the proposed ALGI framework shows potential for practical applications such as genomic selection and genotype imputation in complex livestock genomic regions.

## Figures and Tables

**Table 1:** Summary of the three datasets.

Data Set	Sample Size	No. of SNP
Yeast	3513	28,220
HLA	2504	27,209
Pig SLA	2549	2173

## Data Availability

The data presented in this study are available upon request from the corresponding author.

## Abbreviations

The following abbreviations are used in this manuscript:

BN	Batch normalization
SCDA	Sparse convolutional denoising autoencoder
DL	Deep learning
PCC	Pearson product-moment correlation coefficient
SGD	Stochastic gradient descent

## Appendix A

### Appendix A.1

**Table A1:** Hyperparameters of the ALGI model.

Hyperparameters	Values
Epochs	50/100/140
Batch size	32
Initial learning rate	0.001
Dropout	0.25
Max-pooling size	2
Up-sampling size	2
Filter size	3
Strides	1
Padding	Same
Optimizer	Adam
Activation function	ReLU, except Softmax for the output layer

### Appendix A.2

**Table A2:** Accuracy comparison between different numbers of SNPs on HLA.

Data Set	Model			Missing Rate			
		5%		10%		20%	
		Average	SD	Average	SD	Average	SD
HLA	ALGI	0.9676	1.9 × 10^−4^	0.9651	2.6 × 10^−4^	0.9655	3.5 × 10^−4^
	AE	0.9477	1 × 10^−5^	0.946	1 × 10^−5^	0.9459	1 × 10^−5^
	SCDA	0.9438	6 × 10^−5^	0.9421	1.4 × 10^−4^	0.9416	4.2 × 10^−4^
HLA (2000)	ALGI	0.9451	5 × 10^−5^	0.9429	1.2 × 10^−4^	0.9423	1.4 × 10^−4^
	AE	0.9543	1 × 10^−5^	0.9548	1 × 10^−5^	0.9535	1 × 10^−5^
	SCDA	0.9354	5 × 10^−5^	0.9368	1.6 × 10^−4^	0.9362	1.3 × 10^−4^

### Appendix A.3

**Table A3:** Accuracy comparison between pig chromosomes 7 (SLA) and 1.

Data Set	Model			Missing Rate			
		5%		10%		20%	
		Average	SD	Average	SD	Average	SD
SLA (Chr7)	ALGI	0.9309	1.6 × 10^−4^	0.9306	1.4 × 10^−4^	0.9233	7.2 × 10^−5^
	AE	0.7689	1.2 × 10^−4^	0.7873	2.3 × 10^−4^	0.7788	1.7 × 10^−5^
	SCDA	0.7267	2.4 × 10^−4^	0.7692	3.6 × 10^−4^	0.7682	1.4 × 10^−4^
Chr1	ALGI	0.9466	1.3 × 10^−4^	0.9438	1.2 × 10^−4^	0.9361	2.1 × 10^−4^
	AE	0.7658	1.6 × 10^−4^	0.7789	1.9 × 10^−4^	0.7785	1.3 × 10^−4^
	SCDA	0.7449	1.5 × 10^−4^	0.7779	1.2 × 10^−4^	0.7768	1.9 × 10^−4^
